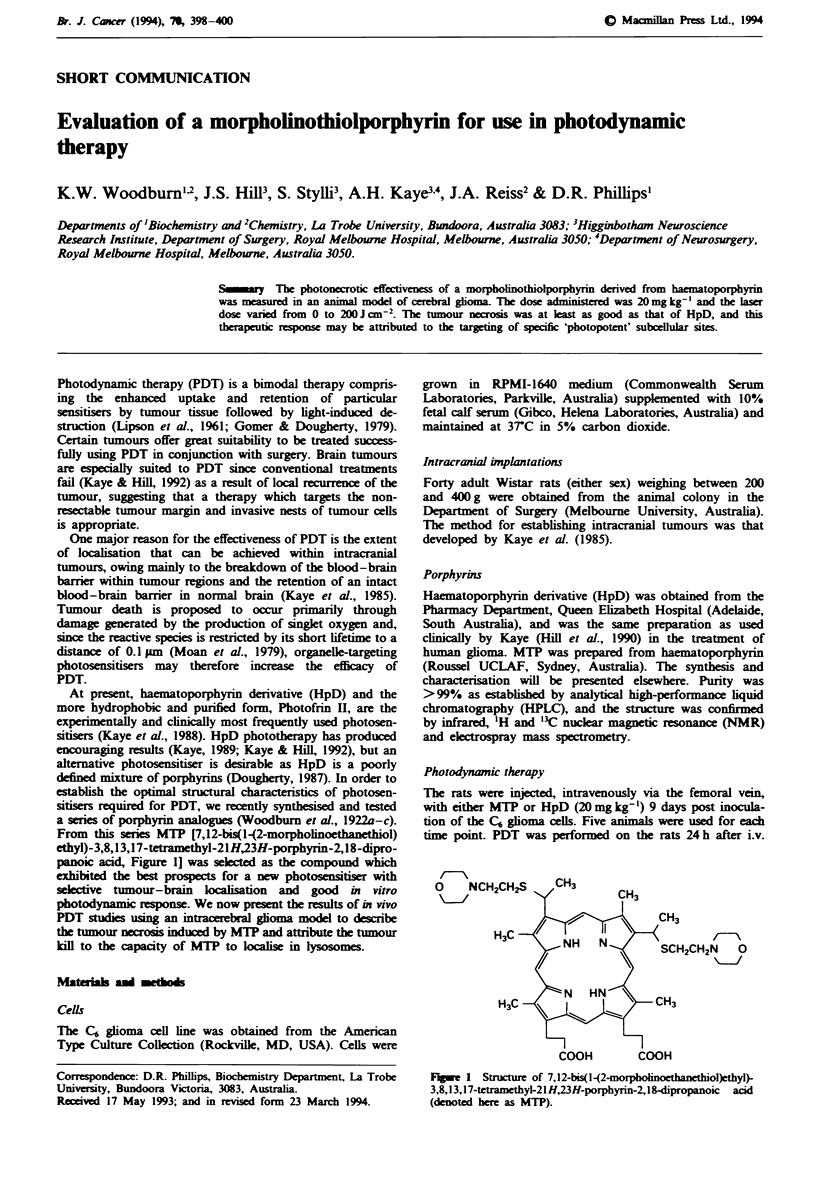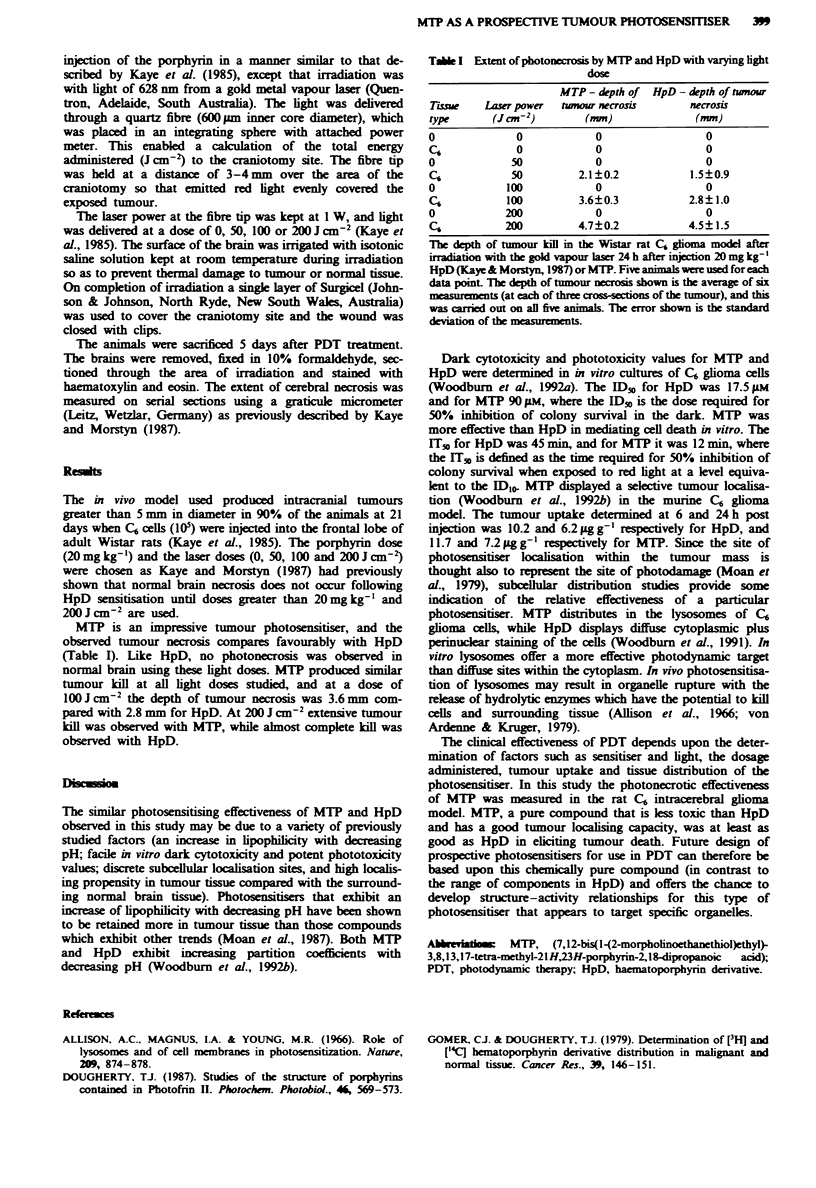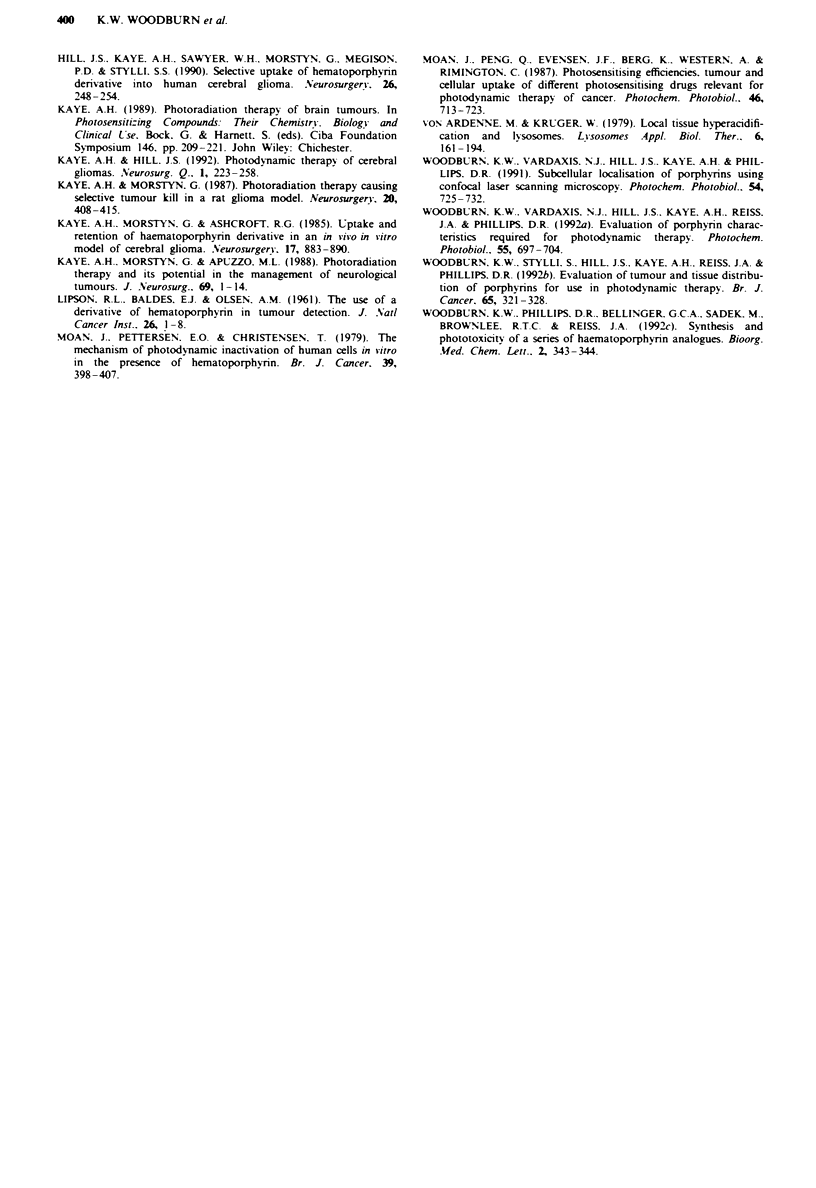# Evaluation of a morpholinothiolporphyrin for use in photodynamic therapy.

**DOI:** 10.1038/bjc.1994.316

**Published:** 1994-09

**Authors:** K. W. Woodburn, J. S. Hill, S. Stylli, A. H. Kaye, J. A. Reiss, D. R. Phillips

**Affiliations:** Department of Biochemistry, La Trobe University, Bundoora, Australia.

## Abstract

The photonecrotic effectiveness of a morpholinothiolporphyrin derived from haematoporphyrin was measured in an animal model of cerebral glioma. The dose administered was 20 mg kg-1 and the laser dose varied from 0 to 200 J cm-2. The tumour necrosis was at least as good as that of HpD, and this therapeutic response may be attributed to the targeting of specific 'photopotent' subcellular sites.


 © Macmillan Press Ltd., 1994

SHORT COMMUNICATION

### Evaluation of a morpholinothiolporphyrin for use in photodynamic therapy

K.W. Woodburn[1,2], J.S. Hill[3], S. Stylli[3], A.H. Kaye[3,4], J.A. Reiss[2] & D.R. Phillips[1]

*Departments of [1]Biochemistry and [2]Chemistry, La Trobe University, Bundoora, Australia 3083; [3]Higginbotham Neuroscience Research Institute, Department of Surgery, Royal Melbourne Hospital, Melbourne, Australia 3050; [4]Department of Neurosurgery, Royal Melbourne Hospital, Melbourne, Australia 3050.*

**Summary** The photonecrotic effectiveness of a morpholinothiolporphyrin derived from haematoporphyrin was measured in an animal model of cerebral glioma. The dose administered was 20 mg $kg^{-1}$ and the laser dose varied from 0 to 200 J $cm^{-2}$. The tumour necrosis was at least as good as that of HpD, and this therapeutic response may be attributed to the targeting of specific 'photopotent' subcellular sites.

Photodynamic therapy (PDT) is a bimodal therapy comprising the enhanced uptake and retention of particular sensitisers by tumour tissue followed by light-induced destruction (Lipson *et al.*, 1961; Gomer & Dougherty, 1979). Certain tumours offer great suitability to be treated successfully using PDT in conjunction with surgery. Brain tumours are especially suited to PDT since conventional treatments fail (Kaye & Hill, 1992) as a result of local recurrence of the tumour, suggesting that a therapy which targets the non-resectable tumour margin and invasive nests of tumour cells is appropriate.

One major reason for the effectiveness of PDT is the extent of localisation that can be achieved within intracranial tumours, owing mainly to the breakdown of the blood-brain barrier within tumour regions and the retention of an intact blood-brain barrier in normal brain (Kaye *et al.*, 1985). Tumour death is proposed to occur primarily through damage generated by the production of singlet oxygen and, since the reactive species is restricted by its short lifetime to a distance of 0.1 µm (Moan *et al.*, 1979), organelle-targeting photosensitisers may therefore increase the efficacy of PDT.

At present, haematoporphyrin derivative (HpD) and the more hydrophobic and purified form, Photofrin II, are the experimentally and clinically most frequently used photosensitisers (Kaye *et al.*, 1988). HpD phototherapy has produced encouraging results (Kaye, 1989; Kaye & Hill, 1992), but an alternative photosensitiser is desirable as HpD is a poorly defined mixture of porphyrins (Dougherty, 1987). In order to establish the optimal structural characteristics of photosensitisers required for PDT, we recently synthesised and tested a series of porphyrin analogues (Woodburn *et al.*, 1922*a–c*). From this series MTP [7,12-bis(1-(2-morpholinoethanethiol) ethyl)-3,8,13,17-tetramethyl-21*H*,23*H*-porphyrin-2,18-dipropanoic acid, Figure 1] was selected as the compound which exhibited the best prospects for a new photosensitiser with selective tumour-brain localisation and good *in vitro* photodynamic response. We now present the results of *in vivo* PDT studies using an intracerebral glioma model to describe the tumour necrosis induced by MTP and attribute the tumour kill to the capacity of MTP to localise in lysosomes.

### Materials and methods

### *Cells*

The $C_6$ glioma cell line was obtained from the American Type Culture Collection (Rockville, MD, USA). Cells were grown in RPMI-1640 medium (Commonwealth Serum Laboratories, Parkville, Australia) supplemented with 10% fetal calf serum (Gibco, Helena Laboratories, Australia) and maintained at 37°C in 5% carbon dioxide.

### *Intracranial implantations*

Forty adult Wistar rats (either sex) weighing between 200 and 400 g were obtained from the animal colony in the Department of Surgery (Melbourne University, Australia). The method for establishing intracranial tumours was that developed by Kaye *et al.* (1985).

### *Porphyrins*

Haematoporphyrin derivative (HpD) was obtained from the Pharmacy Department, Queen Elizabeth Hospital (Adelaide, South Australia), and was the same preparation as used clinically by Kaye (Hill *et al.*, 1990) in the treatment of human glioma. MTP was prepared from haematoporphyrin (Roussel UCLAF, Sydney, Australia). The synthesis and characterisation will be presented elsewhere. Purity was >99% as established by analytical high-performance liquid chromatography (HPLC), and the structure was confirmed by infrared, $^{1}H$ and $^{13}C$ nuclear magnetic resonance (NMR) and electrospray mass spectrometry.

### *Photodynamic therapy*

The rats were injected, intravenously via the femoral vein, with either MTP or HpD (20 mg $kg^{-1}$) 9 days post inoculation of the $C_6$ glioma cells. Five animals were used for each time point. PDT was performed on the rats 24 h after i.v.

**Figure 1** Structure of 7,12-bis(1-(2-morpholinoethanethiol)ethyl)-3,8,13,17-tetramethyl-21*H*,23*H*-porphyrin-2,18-dipropanoic acid (denoted here as MTP).

Correspondence: D.R. Phillips, Biochemistry Department, La Trobe University, Bundoora Victoria, 3083, Australia.
Received 17 May 1993; and in revised form 23 March 1994.

injection of the porphyrin in a manner similar to that described by Kaye *et al.* (1985), except that irradiation was with light of 628 nm from a gold metal vapour laser (Quentron, Adelaide, South Australia). The light was delivered through a quartz fibre (600 μm inner core diameter), which was placed in an integrating sphere with attached power meter. This enabled a calculation of the total energy administered ($J\ cm^{-2}$) to the craniotomy site. The fibre tip was held at a distance of 3–4 mm over the area of the craniotomy so that emitted red light evenly covered the exposed tumour.

The laser power at the fibre tip was kept at 1 W, and light was delivered at a dose of 0, 50, 100 or 200 $J\ cm^{-2}$ (Kaye *et al.*, 1985). The surface of the brain was irrigated with isotonic saline solution kept at room temperature during irradiation so as to prevent thermal damage to tumour or normal tissue. On completion of irradiation a single layer of Surgicel (Johnson & Johnson, North Ryde, New South Wales, Australia) was used to cover the craniotomy site and the wound was closed with clips.

The animals were sacrificed 5 days after PDT treatment. The brains were removed, fixed in 10% formaldehyde, sectioned through the area of irradiation and stained with haematoxylin and eosin. The extent of cerebral necrosis was measured on serial sections using a graticule micrometer (Leitz, Wetzlar, Germany) as previously described by Kaye and Morstyn (1987).

### Results

The *in vivo* model used produced intracranial tumours greater than 5 mm in diameter in 90% of the animals at 21 days when $C_6$ cells ($10^5$) were injected into the frontal lobe of adult Wistar rats (Kaye *et al.*, 1985). The porphyrin dose (20 $mg\ kg^{-1}$) and the laser doses (0, 50, 100 and 200 $J\ cm^{-2}$) were chosen as Kaye and Morstyn (1987) had previously shown that normal brain necrosis does not occur following HpD sensitisation until doses greater than 20 $mg\ kg^{-1}$ and 200 $J\ cm^{-2}$ are used.

MTP is an impressive tumour photosensitiser, and the observed tumour necrosis compares favourably with HpD (Table I). Like HpD, no photonecrosis was observed in normal brain using these light doses. MTP produced similar tumour kill at all light doses studied, and at a dose of 100 $J\ cm^{-2}$ the depth of tumour necrosis was 3.6 mm compared with 2.8 mm for HpD. At 200 $J\ cm^{-2}$ extensive tumour kill was observed with MTP, while almost complete kill was observed with HpD.

**Table I** Extent of photonecrosis by MTP and HpD with varying light dose

| *Tissue type* | *Laser power ($J\ cm^{-2}$)* | *MTP – depth of tumour necrosis (mm)* | *HpD – depth of tumour necrosis (mm)* |
|---|---|---|---|
| 0 | 0 | 0 | 0 |
| $C_6$ | 0 | 0 | 0 |
| 0 | 50 | 0 | 0 |
| $C_6$ | 50 | 2.1±0.2 | 1.5±0.9 |
| 0 | 100 | 0 | 0 |
| $C_6$ | 100 | 3.6±0.3 | 2.8±1.0 |
| 0 | 200 | 0 | 0 |
| $C_6$ | 200 | 4.7±0.2 | 4.5±1.5 |

The depth of tumour kill in the Wistar rat $C_6$ glioma model after irradiation with the gold vapour laser 24 h after injection 20 $mg\ kg^{-1}$ HpD (Kaye & Morstyn, 1987) or MTP. Five animals were used for each data point. The depth of tumour necrosis shown is the average of six measurements (at each of three cross-sections of the tumour), and this was carried out on all five animals. The error shown is the standard deviation of the measurements.

Dark cytotoxicity and phototoxicity values for MTP and HpD were determined in *in vitro* cultures of $C_6$ glioma cells (Woodburn *et al.*, 1992*a*). The $ID_{50}$ for HpD was 17.5 μM and for MTP 90 μM, where the $ID_{50}$ is the dose required for 50% inhibition of colony survival in the dark. MTP was more effective than HpD in mediating cell death *in vitro*. The $IT_{50}$ for HpD was 45 min, and for MTP it was 12 min, where the $IT_{50}$ is defined as the time required for 50% inhibition of colony survival when exposed to red light at a level equivalent to the $ID_{10}$. MTP displayed a selective tumour localisation (Woodburn *et al.*, 1992*b*) in the murine $C_6$ glioma model. The tumour uptake determined at 6 and 24 h post injection was 10.2 and 6.2 $\mu g\ g^{-1}$ respectively for HpD, and 11.7 and 7.2 $\mu g\ g^{-1}$ respectively for MTP. Since the site of photosensitiser localisation within the tumour mass is thought also to represent the site of photodamage (Moan *et al.*, 1979), subcellular distribution studies provide some indication of the relative effectiveness of a particular photosensitiser. MTP distributes in the lysosomes of $C_6$ glioma cells, while HpD displays diffuse cytoplasmic plus perinuclear staining of the cells (Woodburn *et al.*, 1991). *In vitro* lysosomes offer a more effective photodynamic target than diffuse sites within the cytoplasm. *In vivo* photosensitisation of lysosomes may result in organelle rupture with the release of hydrolytic enzymes which have the potential to kill cells and surrounding tissue (Allison *et al.*, 1966; von Ardenne & Kruger, 1979).

The clinical effectiveness of PDT depends upon the determination of factors such as sensitiser and light, the dosage administered, tumour uptake and tissue distribution of the photosensitiser. In this study the photonecrotic effectiveness of MTP was measured in the rat $C_6$ intracerebral glioma model. MTP, a pure compound that is less toxic than HpD and has a good tumour localising capacity, was at least as good as HpD in eliciting tumour death. Future design of prospective photosensitisers for use in PDT can therefore be based upon this chemically pure compound (in contrast to the range of components in HpD) and offers the chance to develop structure–activity relationships for this type of photosensitiser that appears to target specific organelles.

### Discussion

The similar photosensitising effectiveness of MTP and HpD observed in this study may be due to a variety of previously studied factors (an increase in lipophilicity with decreasing pH; facile *in vitro* dark cytotoxicity and potent phototoxicity values; discrete subcellular localisation sites, and high localising propensity in tumour tissue compared with the surrounding normal brain tissue). Photosensitisers that exhibit an increase of lipophilicity with decreasing pH have been shown to be retained more in tumour tissue than those compounds which exhibit other trends (Moan *et al.*, 1987). Both MTP and HpD exhibit increasing partition coefficients with decreasing pH (Woodburn *et al.*, 1992*b*).

**Abbreviations:** MTP, (7,12-bis(1-(2-morpholinoethanethiol)ethyl)-3,8,13,17-tetra-methyl-21*H*,23*H*-porphyrin-2,18-dipropanoic acid); PDT, photodynamic therapy; HpD, haematoporphyrin derivative.

### References

ALLISON, A.C., MAGNUS, I.A. & YOUNG, M.R. (1966). Role of lysosomes and of cell membranes in photosensitization. *Nature*, **209**, 874–878.

DOUGHERTY, T.J. (1987). Studies of the structure of porphyrins contained in Photofrin II. *Photochem. Photobiol.*, **46**, 569–573.

GOMER, C.J. & DOUGHERTY, T.J. (1979). Determination of [$^3$H] and [$^{14}$C] hematoporphyrin derivative distribution in malignant and normal tissue. *Cancer Res.*, **39**, 146–151.

HILL, J.S., KAYE, A.H., SAWYER, W.H., MORSTYN, G., MEGISON, P.D. & STYLLI, S.S. (1990). Selective uptake of hematoporphyrin derivative into human cerebral glioma. *Neurosurgery*, **26**, 248-254.

KAYE, A.H. (1989). Photoradiation therapy of brain tumours. In *Photosensitizing Compounds: Their Chemistry, Biology and Clinical Use*, Bock, G. & Harnett, S. (eds). Ciba Foundation Symposium 146, pp. 209-221. John Wiley: Chichester.

KAYE, A.H. & HILL, J.S. (1992). Photodynamic therapy of cerebral gliomas. *Neurosurg. Q.*, **1**, 223-258.

KAYE, A.H. & MORSTYN, G. (1987). Photoradiation therapy causing selective tumour kill in a rat glioma model. *Neurosurgery*, **20**, 408-415.

KAYE, A.H., MORSTYN, G. & ASHCROFT, R.G. (1985). Uptake and retention of haematoporphyrin derivative in an *in vivo in vitro* model of cerebral glioma. *Neurosurgery*, **17**, 883-890.

KAYE, A.H., MORSTYN, G. & APUZZO, M.L. (1988). Photoradiation therapy and its potential in the management of neurological tumours. *J. Neurosurg.*, **69**, 1-14.

LIPSON, R.L., BALDES, E.J. & OLSEN, A.M. (1961). The use of a derivative of hematoporphyrin in tumour detection. *J. Natl Cancer Inst.*, **26**, 1-8.

MOAN, J., PETTERSEN, E.O. & CHRISTENSEN, T. (1979). The mechanism of photodynamic inactivation of human cells *in vitro* in the presence of hematoporphyrin. *Br. J. Cancer*, **39**, 398-407.

MOAN, J., PENG, Q., EVENSEN, J.F., BERG, K., WESTERN, A. & RIMINGTON, C. (1987). Photosensitising efficiencies, tumour and cellular uptake of different photosensitising drugs relevant for photodynamic therapy of cancer. *Photochem. Photobiol.*, **46**, 713-723.

VON ARDENNE, M. & KRUGER, W. (1979). Local tissue hyperacidification and lysosomes. *Lysosomes Appl. Biol. Ther.*, **6**, 161-194.

WOODBURN, K.W., VARDAXIS, N.J., HILL, J.S., KAYE, A.H. & PHILLIPS, D.R. (1991). Subcellular localisation of porphyrins using confocal laser scanning microscopy. *Photochem. Photobiol.*, **54**, 725-732.

WOODBURN, K.W., VARDAXIS, N.J., HILL, J.S., KAYE, A.H., REISS, J.A. & PHILLIPS, D.R. (1992*a*). Evaluation of porphyrin characteristics required for photodynamic therapy. *Photochem. Photobiol.*, **55**, 697-704.

WOODBURN, K.W., STYLLI, S., HILL, J.S., KAYE, A.H., REISS, J.A. & PHILLIPS, D.R. (1992*b*). Evaluation of tumour and tissue distribution of porphyrins for use in photodynamic therapy. *Br. J. Cancer*, **65**, 321-328.

WOODBURN, K.W., PHILLIPS, D.R., BELLINGER, G.C.A., SADEK, M., BROWNLEE, R.T.C. & REISS, J.A. (1992*c*). Synthesis and phototoxicity of a series of haematoporphyrin analogues. *Bioorg. Med. Chem. Lett.*, **2**, 343-344.